# Genome sequence of parvovirus from budgerigar (*Melopsittacus undulatus*)

**DOI:** 10.1128/MRA.00453-23

**Published:** 2023-10-09

**Authors:** Dominika Kadlečková, Michal Vinkler, Ruth Tachezy, Martina Saláková

**Affiliations:** 1 Department of Genetics and Microbiology, Faculty of Science BIOCEV, Charles University, Vestec, Czech Republic; 2 Department of Zoology, Faculty of Science, Charles University, Prague, Czech Republic; Katholieke Universiteit Leuven, Leuven, Belgium

**Keywords:** parvovirus, budgerigar, *Melopsittacus undulatus*, NGS

## Abstract

Here, we report a parvovirus genome identified in *Melopsittacus undulatus*. The genome is 4,547 bp long and codes for two major open reading frames (ORFs): the non-structural replicase protein 1 (NS1) and the structural capsid gene (VP1). Phylogenetic analysis shows that this virus belongs to the genus *Chaphamaparvovirus*.

## ANNOUNCEMENT

Parvoviruses are known to infect the gastrointestinal tracts of domestic fowl as well as urban wild birds (1, 2). So far, there are only a few metagenomics/metatranscriptomic studies exploring the virome in birds (3). Here, we characterize a parvovirus genome found in a healthy female budgerigar (*Melopsittacus undulatus*) collected in September 2021 in Czechia: Přišimasy (Kolín).

**Fig 1:**
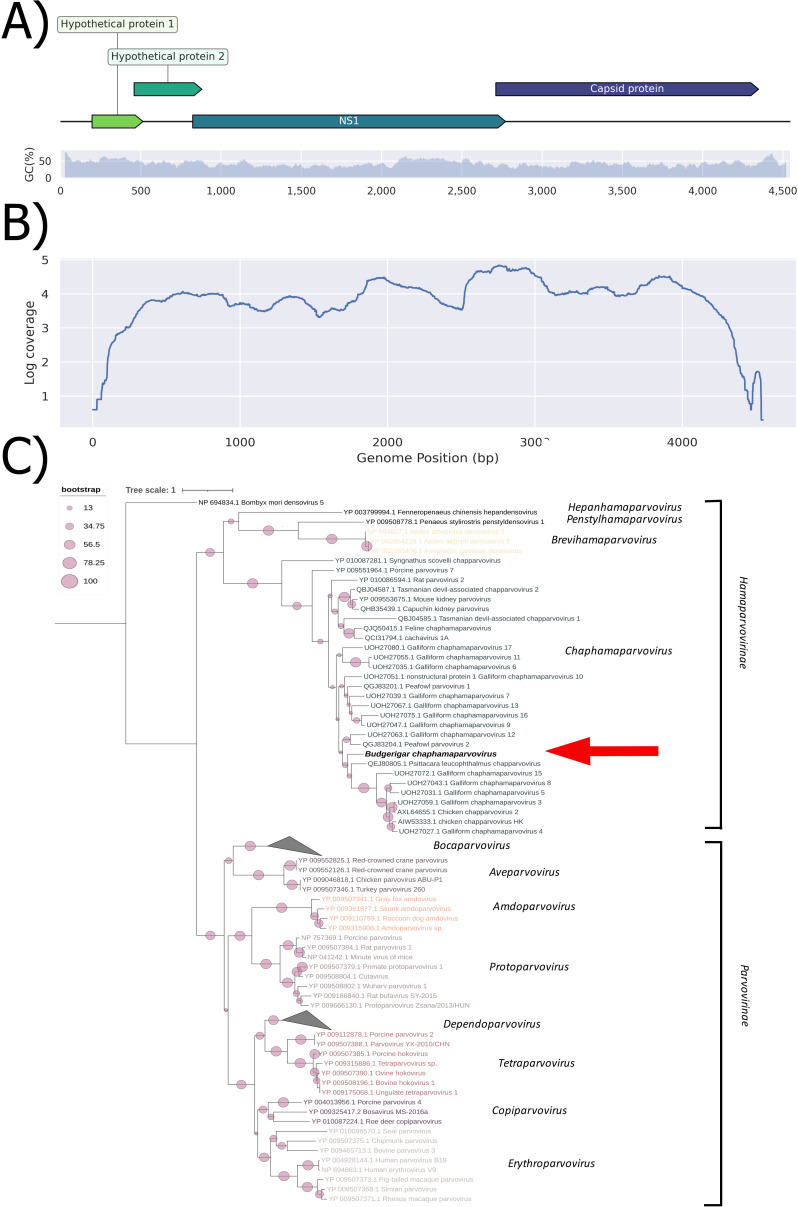
(A) Scheme of the genome of Budgerigar chaphamaparvovirus with GC content displayed below. (**B**) Depth coverage of the genome. (**C**) Phylogenetic tree, amino acid sequences of complete NS1 aligned with MAFFT v7.520 (11), trimmed with trimAl v1.4.1 (12), the best model was determined with ModelTest-NG v0.1.7 (13), and a tree with bootstraps (100) was created with PhyML v3.3.20220408 (14) and visualized in iTOL (15).

Fresh feces were frozen at −80°C. The sample was homogenized in 500 µL of 1×phosphate-buffered saline (PBS) and processed according to NetoVir protocol (4). Briefly, the sample was centrifuged, filtered, and treated with nucleases, and nucleic acids enclosed in viral capsids were extracted using QIAamp Viral RNA Mini Kit (Qiagen, Hilden, Germany). Nucleic acids were amplified with a modified WTA2 protocol (Sigma-Aldrich, St. Louis, MO, USA); see NetoVIR protocol (4). The library was prepared with Nextera XT Library Preparation Kit (Illumina, San Diego, CA, USA). The sequencing was performed on the NextSeq 500 Illumina platform (2 × 150 bp paired-end, Laboratory of Genomics and Bioinformatics, IMG, Czechia). The quality of the reads was checked using FastQC v0.11.9 (Babraham Bioinformatics, Babraham, UK). Trimming was done with Trimmomatic v0.32 (5) with settings to trim adapters from WTA2 and NexteraXT with ILLUMINACLIP, HEADCROP:19, LEADING:15, TRAILING:15, SLIDINGWINDOW:4:20, and MINLEN:50. Trimmed sequences were assembled using SPAdes v3.15.5 (6) with settings --meta, -k 21, 33, 55, and 77. Reads were mapped back to contigs with bwa-mem2 v2.2.1 (7), and coverage was extracted with CoverM v0.6.1 (https://github.com/wwood/CoverM). Contigs were compared to the nr database (NCBI) downloaded on 8 August 2021 using DIAMOND v2.0.15 (8) with a sensitive setting. Settings not specified were used with default parameters. Most viral reads (83% of all viral reads, 3% of all reads, 386,330 reads out of 13,709,189, and ~3% of all reads) were mapped to one contig identified as the parvovirus genome (Fig. 1B). The sequence was blasted with BLASTx (9) against the nr database and showed the highest similarity to avian chapparvovirus (54.04%, QKX49056.1). The length of the assembled contig was 4,547 bp, with a G-C content of 45.0%. The first major ORF predicted using Prodigal (10) encoded a non-structural protein 1 (NS1; 650 aa) that plays a role in the initiation of viral replication. The predicted NS1 protein shared 54.6% amino acid identity with NS1 of avian chapparvovirus (QKX49056.1). The second major ORF encodes a capsid protein with a length of 546 aa with the highest similarity (48.83%) to the capsid protein of the blue-and-yellow macaw (*Ara ararauna*) Chaphamaparvovirus (QTE04011.1). Two short ORFs with hypothetical proteins 1 (106 aa) and 2 (141 aa) were closest to Phoenicopteridae parvo-like hybrid virus (QTE03742.1) and *Chufflevirus* sp. (QSH48278.1) with an identity of 49.64% and 62.92%, respectively. The genome organization is shown in Fig. 1A.

For the phylogenetic analysis (Fig. 1C), amino acid sequences of NS1 from several genera of *Parvovirinae* and *Hamaparvovirinae* were collected from NCBI. A representative of *Densovirinae* was used as an outgroup. The result shows that the virus, tentatively named Budgerigar chaphamaparvovirus, belongs to the subfamily *Hamaparvovirinae* and genus *Chaphamaparvoviruses* (16).

## Data Availability

Sequencing reads have been deposited into Sequence Read Archive (SRA) under the accession number SRR21387183 (BioProject PRJNA875655). The genome of parvovirus was deposited into NCBI GenBank under accession number OP359044.
